# Cold Adaptation Mechanisms of a Snow Alga *Chlamydomonas nivalis* During Temperature Fluctuations

**DOI:** 10.3389/fmicb.2020.611080

**Published:** 2021-01-11

**Authors:** Zhao Peng, Gai Liu, Kaiyao Huang

**Affiliations:** ^1^Key Laboratory of Algal Biology, Institute of Hydrobiology, Chinese Academy of Sciences, Wuhan, China; ^2^University of Chinese Academy of Sciences, Beijing, China

**Keywords:** cold adaptation, RNA-seq, horizontal gene transfer, *Chlamydomonas reinhardtii*, snow algae

## Abstract

Cold environments, such as glaciers and alpine regions, constitute unique habitats for organisms living on Earth. In these harsh ecosystems, snow algae survive, florish, and even become primary producers for microbial communities. How the snow algae maintain physiological activity during violent ambient temperature changes remains unsolved. To explore the cold adaptation mechanisms of the unicellular snow alga *Chlamydomonas nivalis*, we compared its physiological responses to a model organism from the same genus, *Chlamydomonas reinhardtii*. When both cell types were exposed to a shift from 22°C to 4°C, *C. nivalis* exhibited an apparent advantage in cold tolerance over *C. reinhardtii*, as *C. nivalis* had both a higher growth rate and photosynthetic efficiency. To determine the cold tolerance mechanisms of *C. nivalis*, RNA sequencing was used to compare transcriptomes of both species after 1 h of cold treatment, mimicking temperature fluctuations in the polar region. Differential expression analysis showed that *C. nivalis* had fewer transcriptomic changes and was more stable during rapid temperature decrease relative to *C. reinhardtii*, especially for the expression of photosynthesis related genes. Additionally, we found that transcription in *C. nivalis* was precisely regulated by the cold response network, consisting of at least 12 transcription factors and 3 RNA-binding proteins. Moreover, genes participating in nitrogen metabolism, the pentose phosphate pathway, and polysaccharide biosynthesis were upregulated, indicating that increasing resource assimilation and remodeling of metabolisms were critical for cold adaptation in *C. nivalis*. Furthermore, we identified horizontally transferred genes differentially expressed in *C. nivalis*, which are critical for cold adaptation in other psychrophiles. Our results reveal that *C. nivalis* adapts rapid temperature decrease by efficiently regulating transcription of specific genes to optimize resource assimilation and metabolic pathways, providing critical insights into how snow algae survive and propagate in cold environments.

## Introduction

Cold environments, where the temperature remains persistently below or close to the freezing point of 0°C, occupy more than 80% of the biosphere on Earth, comprising both terrestrial ecosystems and aquatic regions, such as deep oceans and subglacial lakes (Feller and Gerday, 2003; Morgan-Kiss et al., 2006). Most cold terrestrial habitats, including alpine regions and polar glaciers, are covered permanently or seasonally by snow (Margesin and Miteva, 2011). The spread of land plants and mammals is limited in such harsh niches. However, psychrophiles survive and thrive on the snowfield. Psychrophiles can include bacteria, algae, fungi, and protozoa, Snow algae, organisms known for producing colorful patches on snowfields, act as the primary producer by providing energy and nutrients for whole psychrophilic biomes. Moreover, these photoautotrophic species can decrease the albedo of the snow in alpine, Antarctic, and Arctic areas, leading to an increase in the melting rate of snow and ice, and ultimately accelerating the retreat of glaciers (Lutz et al., 2016; Davey et al., 2019).

Snow algae belong to the phylum Chlorophyta, primarily consisting of the genera *Chloromonas* and *Chlamydomonas* (Hoham and Duval, 2001). The life cycle of snow algae mainly comprises two periods with distinct cell shapes (Hoham and Blinn, 1979). In winter, the red spherical cyst spores survive beneath the snow. In late spring or early summer, snow algae flourish as green motile vegetative cells in the melting snow (Remias et al., 2005). When snow vanishes in late summer, the algae cells become dehydrated and are exposed to direct sunlight. Thus, snow algae must adapt to extremely high photosynthetically active radiation, ultraviolet radiation, desiccation, nutrition restriction, and especially low temperatures (Remias et al., 2005).

Low temperatures constitute a unique challenge to photoautotrophic organisms. At low temperatures, excess excitation energy is generated, potentially damaging photosynthetic apparatus, by over-reduction of the plastoquinone pool and excessive reactive oxygen species (Huner et al., 2013). *Chlamydomonas nivalis* UTEX 2824 is a snow alga isolated from the Sierra Nevada Mountains in California that has been used as a research model to study the behavior of snow algae (Thomas, 1972). Snow algae, including *C. nivalis*, have been found to synthesize abundant special chemicals, such as carotenoids and extracellular polysaccharides, to facilitate cold adaptation (Muller et al., 1998; De Maayer et al., 2014). Another study showed that the change of photosynthetic electron transfer and thylakoid lipid composition was temperature dependent in *C. nivalis* (Lukes et al., 2014). Recently, a study showed that *C. nivalis* UTEX 2824 can reduce the light-harvesting ability of photosystem II and induce cyclic electron transfer around photosystem I to avoid the decline of photosynthetic efficiency at low temperatures. By regulating photosynthesis, *C. nivalis* UTEX 2824 not only produces ATP for cellular growth, but also eliminates the damage caused by excess excitation (Zheng et al., 2020). However, how *C. nivalis* UTEX 2824 acclimates to cold stress, especially abrupt temperature decrease, remains largely unclear.

Recently, genomic sequencing has advanced the understanding of cold adaptation mechanisms in snow or ice algae. For example, *Coccomyxa subellipsoidea* C-169, the first polar green alga to have its genome sequenced, increases the expression of lipid biosynthesis genes to maintain membrane fluidity at low temperatures, and produces antifreeze proteins and exopolysaccharides to reduce the freezing point in the cytoplasm (Blanc et al., 2012). *Fragilariopsis cylindrus* is a cold-adapted diatom that lives in the Southern Ocean and possesses highly divergent alleles transcribed dynamically in response to highly variable environmental conditions (Mock et al., 2017). *Chlorella vulgaris* NJ-7 is a psychrotolerant alga living in Antarctica. Compared to its mesophilic relative *C. vulgaris* UTEX 259, NJ-7 expresses high levels of nitrate reductases and cryoprotective late embryogenesis abundant proteins to compensate for their low activity at lower temperatures (Wang et al., 2020). More recently, sequencing of the ice alga *Chlamydomonas* ICE-L indicated that an expansion of ice-binding protein (IBP) gene families had occurred through horizontal gene transfer (HGT) (Zhang et al., 2020). Beyond genomic sequencing, transcriptomic analyses have also been applied to understand the mechanisms of cold adaptation in snow algae. The expression of genes related to lipid metabolism and nitrogen metabolism were reported to increase in *Chlamydomonas* ICE-L (Liu C. et al., 2016). Transcriptional profiling of the arctic algae *Chlorella-*Arc showed that photosynthesis, carotenoid biosynthesis, and the pentose phosphate pathway, were involved in resisting thermal/cold stress (Song et al., 2020).

Gene regulation at the transcriptional level is one of the most important responses to environmental stresses. To resist and acclimate to stress, the transcription of many genes is regulated, which can be divided into regulatory genes upstream and functional genes downstream (Singh and Laxmi, 2015). In the mesophilic green alga *Chlamydomonas reinhardtii*, which has well-established gene annotations (Rymarquis et al., 2005; Merchant et al., 2007; Blaby et al., 2014), 3,471 genes were differentially expressed after 1 h cold exposure (Li et al., 2020). To survive at low temperatures, *C. nivalis* may possess unique cold adaptation mechanisms, as reflected in different transcriptional changes of regulatory and functional genes during cold exposure compared with *C. reinhardtii*. So we conducted a comparative transcriptomic analysis between *C. nivalis* and *C. reinhardtii*, to clarify the differences of transcriptional changes upon cold treatment between the two species, and identify the unique regulatory network upstream and biological pathways downstream in *C. nivalis*. Our study reveals that *C. nivalis* adjusts to temperature fluctuations through specific transcription factors and RNA-binding proteins that optimizes resource assimilation and metabolic pathways.

## Materials and Methods

### Strains and Culture Conditions

*Chlamydomonas nivalis* UTEX 2824 was obtained from the Culture Collection of Algae at the University of Texas at Austin, and grown in liquid or solid (1.5% agar) Bold 1NV (B1NV) medium (recipe details provided by the Culture Collection of Algae at the University of Texas, Austin, TX, United States). The *C. reinhardtii* CC-124 (*Chlamydomonas* Resource Center, St. Paul, MN, United States) strain was used in this study, and grown in liquid or solid (1.5% agar) Tris-acetate-phosphate (TAP) medium (Harris, 1989). Before cold treatment at 4°C, cells of both algae were cultured at 22°C and agitated at 120 rpm under continuous light of ∼50 μmol photon m^–1^ s^–2^. Cell concentrations were determined using a hemocytometer.

### *F_*V*_/F_*M*_* Measurements

*F_*V*_/F_*M*_* was measured using a multiple excitation wavelength modulated chlorophyll fluorometer (Heinz Walz GmbH, Effeltrich, Germany). Cells at a concentration of 10^6^ mL^–1^ were transferred to a cuvette and dark-adapted for 15 min before performing *F_*V*_/F_*M*_* measurements.

### Extraction and Quantification of EPS

Exopolysaccharides (EPS) were extracted using the cation exchange resin (CER) method (Zheng et al., 2019). The algae cells were grown at 22°C until the mid-log phase and transferred to 4°C for 3 days, with negative controls kept growing at 22°C for 3 days. Cells were collected by centrifuging at 13,000 × *g* for 10 min. The pellet was washed twice with fresh culture medium and resuspended in extraction buffer solution (2 mM Na_3_PO_4_, 4 mM NaH_2_PO_4_, 9 mM NaCl, 1 mM KCl, pH = 7, 0.3 g mL^–1^). The solution was then mixed with 2.5 g CER per gram of cell biomass and agitated at 120 rpm at 4°C overnight. The mixture was centrifuged at 20,000 × *g* for 20 min. The supernatant rich in EPS was used for quantification.

The quantification of EPS was performed using the phenol-sulfuric acid method. First, 1 mL of the sample was transferred into a tube, placed on ice, and mixed with 0.5 mL phenol. Then, 0.5 mL sulfuric acid was slowly added dropwise to avoid a sharp heat release. The mixture was placed in boiling water for 20 min and cooled for 10 min. The absorbance of the mixture at 490 nm was measured to determine the concentration of EPS. To establish a standard curve, glucose solutions diluted to final concentrations of 10–100 μg mL^–1^ were treated as above to determine the relationship between absorbance and concentration.

### RNA Extraction

*Chlamydomonas nivalis* UTEX 2824 and *C. reinhardtii* CC-124 cells were grown at 22°C until the mid-log phase and transferred to 4°C for 1 h. Cultures were collected before (0 h) and after (1 h) the cold treatment and centrifuged at 4, 000 × g for 5 min. For *C. reinhardtii* samples, the pellet was resuspended in 5 mL TRIzol (Thermo Fisher, Waltham, MA, United States), and RNA extraction was performed according to the manufacturer’s instructions. For *C. nivalis* samples with thicker cell walls, the pellet was ground with liquid nitrogen to a fine powder and dissolved in CTAB lysis buffer (2% CTAB, 1 M Tris-HCl pH 8.0, 100 mM EDTA pH 8.0, 5 M NaCl) with 10% β-mercaptoethanol. The solution was added with the same volume of chloroform–isopentanol (24:1) and centrifuged at 12,000 × *g* for 10 min at 4°C to collect the aqueous phase. This extraction step was repeated twice. Then, the aqueous phase was mixed with a one-fourth volume of LiCl solution and kept at −20°C for 2 h. After centrifugation at 10,000 × *g* for 30 min at 4°C, the pellet was washed twice with 75% ethanol and resuspended in H_2_O containing 0.1% diethyl pyrocarbonate. The concentration of RNA was measured using a NanoDrop 2000 spectrophotometer (Thermo Fisher).

### Quantitative Reverse Transcription PCR

*Chlamydomonas nivalis* RNA samples were collected through RNA extraction as detailed above. Primer pairs used in this procedure (Supplementary Table 1) were designed for 20 randomly selected differentially expressed genes (DEGs). RNA was reverse transcribed into cDNA using a First Strand cDNA Synthesis Kit (Thermo Fisher) according to the manufacturer’s manual. Using cDNA, the primer pairs and LightCycler 480 SYBR Green (Roche, Basel, Switzerland), Ct values were determined using quantitative real-time PCR on the Applied Biosystems 7500 Fast real-time PCR system (Thermo Fisher). The *CBLP* amplification product was used as an internal standard, and relative fold changes between 0 h and 1 h samples were calculated for each gene, based on the 2^−ΔΔCt^ method (Livak and Schmittgen, 2001).

### RNA Sequencing

RNA sequencing was performed in cooperation with Annoroad Gene Technology (Beijing, China). The cDNA library was prepared and processed using the Illumina TruSeq stranded RNA kit (Illumina, San Diego, CA, United States). After sequencing on the Illumina HiSeq 2000 sequencing system (Illumina), approximately 20 million paired-end reads with the length of 150 bases were produced for each sample. Quality control, low-quality filtering and adapter cutting were performed sequentially to produce clean reads for further processing.

### Transcriptome *de novo* Assembly

To assemble the transcriptome of *C. reinhardtii* and *C. nivalis de novo*, we used Trinity software on the clean reads from the 0 h and 1 h groups separately without using a reference genome (Grabherr et al., 2011). To compare the assembled transcriptomes from different groups, quality assessment scripts within Trinity helped to determine descriptive statistics of the transcriptome, including transcript number, transcript length, and the proportion of transcripts that appeared to be full-length.

### RNA-Seq Data Processing

The clean reads from different samples were aligned to the genome of the corresponding species using STAR software (Dobin et al., 2013). For *C. reinhardtii*, version 5.5 genome data were acquired from Phytozome^1^. For *Chlamydomonas nivalis*, a genome draft was used. Based on the alignment results, the reads mapped to each gene were counted using the featureCounts software (Liao et al., 2014). A Pearson correlation analysis was used to confirm that the two biological replicates were well correlated (Supplementary Figure 1). Differential expression between 0 h and 1 h samples in either *C. nivalis* or *C. reinhardtii* was estimated using the R package DESeq2 (Love et al., 2014). The genes with more than 10 counts in at least two samples were selected first to generate a gene expression matrix. According to the matrix, fold changes and FDR-adjusted *P*-values were calculated for each gene, and DEGs were identified, using the threshold as fold change > 2^0.5^ and FDR-adjusted *P*-value < 0.01.

### Transcriptome Analysis

Annotation and classification of genes was based on the MapMan database (Thimm et al., 2004) and Kyoto Encyclopedia of Genes and Genomes (KEGG) database (Kanehisa, 2000). The enrichment analysis was performed by comparing the number of genes annotated to a function group in the input list, with the number of genes annotated to the group in the entire background set using Fisher’s exact test (Carbon et al., 2019). To identify transcription factors, a prediction function was applied in the PlantTFDB database (Jin et al., 2017). Protein–protein interactions, which are either known or predicted, and include direct (physical) and indirect (functional) associations, were predicted using the STRING database (Szklarczyk et al., 2017). Scatter plots, bar charts, and bubble charts were drawn with the help of the R package ggplot2 (Wickham and Chang, 2008). GO topology graphs were drawn with the help of the R package topGO (Alexa and Rahnenfuhrer, 2010). To identify candidate genes that might originate from HGT, we used Blastp to annotate the DEGs in *C. nivalis* with a non-redundant database from NCBI^2^. We selected genes that had a top hit to sequences from bacteria and fungi, and then removed genes without an assigned function. The filtered genes were listed as candidate horizontally transferred genes. To certify the candidate genes, we first retrieved the top 10 hits of Blastp result for each gene, and then carried on multi-aligning using MUSCLE software (Edgar, 2004). The alignment results were trimmed using trimAl software to exclude unnecessary regions (Capella-Gutierrez et al., 2009), and then processed to generate maximum likelihood phylogenetic trees using IQ-TREE software, setting the number of bootstrap replicates as 1000 (Nguyen et al., 2015). The trees were drawn using iTOL (Letunic and Bork, 2007). We then checked the clade and support value of each candidate gene in its phylogenetic tree for certification.

## Results

### Cold Tolerance of *C. nivalis* UTEX 2824 and *C. reinhardtii* CC-124

**FIGURE 1:**
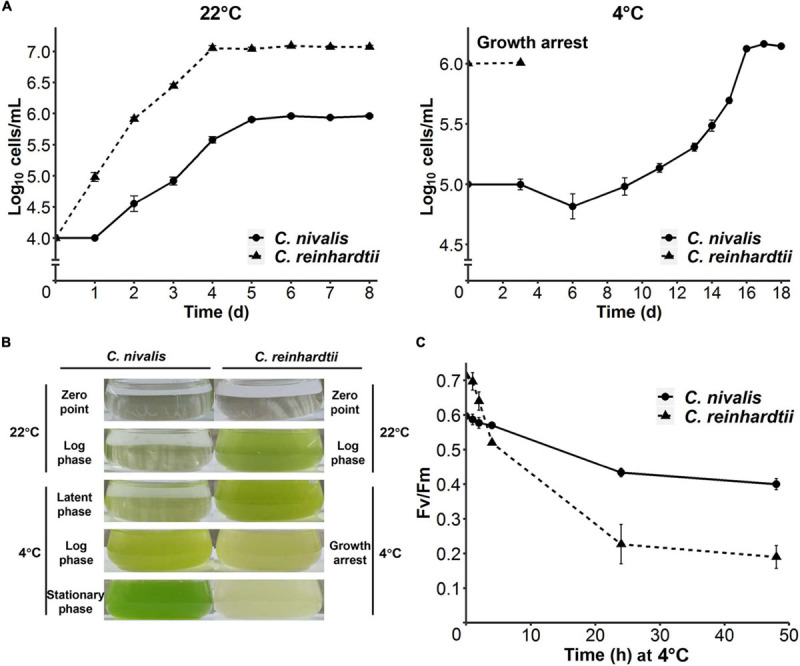
Divergent cold acclimation capacity between *C. nivalis* and *C. reinhardtii.* **(A)** Growth curves of *C. nivalis* and *C. reinhardtii* at 22°C and 4°C, measured everyday (22°C) or every 2–3 days (4°C). For 4°C growth curve, algae cells at 0 h point had been cultured at 22°C until mid-log phase was reached. The error bars represent standard deviations of three biological replicates. **(B)** Comparison between different culture states of *C. nivalis* and *C. reinhardtii*. Photographs show culture states at five phases of growth for *C. nivalis*, which was grown at 22°C until mid-log phase and then transferred to 4°C. For each phase, the culture state of *C. reinhardtii* is shown on the right for comparison. **(C)** *F*_*V*_/*F*_*M*_ measurement of *C. nivalis* and *C. reinhardtii* upon cold treatment. Cells at log phase were transferred to 4°C condition, and then *F_*V*_/F_*M*_* was measured after 1, 2, 4, 24, and 48 h. The error bars represent standard deviations of three biological replicates.

To compare the cold tolerance of *C. nivalis* UTEX 2824 with the mesophilic species *C. reinhardtii* CC-124, both algal cells were first grown at 22°C with an initial concentration of 10^4^ cells mL^–1^. Both species reached their stationary phase after 4 days at 22°C. *C. nivalis* had a rest period of 24 h after inoculation, grew with a doubling time of 15 h, and the density of the stationary phase was only one-tenth of that in *C. reinhardtii*, probably due to the difference of culture medium (Figure 1A, left panel). After transferring the mid-log phase growing cells of UTEX 2824 and CC-124 from 22°C to 4°C, *C. reinhardtii* stopped growth after 3 days, but *C. nivalis* continued to grow after a latent phase, accelerated the growth rate to a doubling time of 1.5 days, and reached a similar concentration as at 22°C (Figure 1A, right panel and Figure 1B). In summary, these results confirmed that *C. nivalis* UTEX 2824 is a psychrotolerant species, while *C. reinhardtii* CC-124 is a mesophilic species that fails to survive at temperatures as low as 4°C.

We also measured the maximum chlorophyll photochemical efficiency of photosystem II (*F_*V*_/F_*M*_*) in *C. nivalis* and *C. reinhardtii* after the transfer from 22°C to 4°C. Although *F_*V*_/F_*M*_* decreased notably for the first 5 h in both species, the *F_*V*_/F_*M*_* of *C. nivalis* remained at 0.4 after 24 h, while that of the mesophilic species *C. reinhardtii* plummeted to 0.2 within the same time. These results suggest that the activity of photosystem II was damaged in *C. reinhardtii* by the low temperature, but *C. nivalis* could sustain the activity at a relatively lower efficiency (Figure 1C).

### DEGs Induced by Cold Stress in *C. nivalis* and *C. reinhardtii*

**FIGURE 2:**
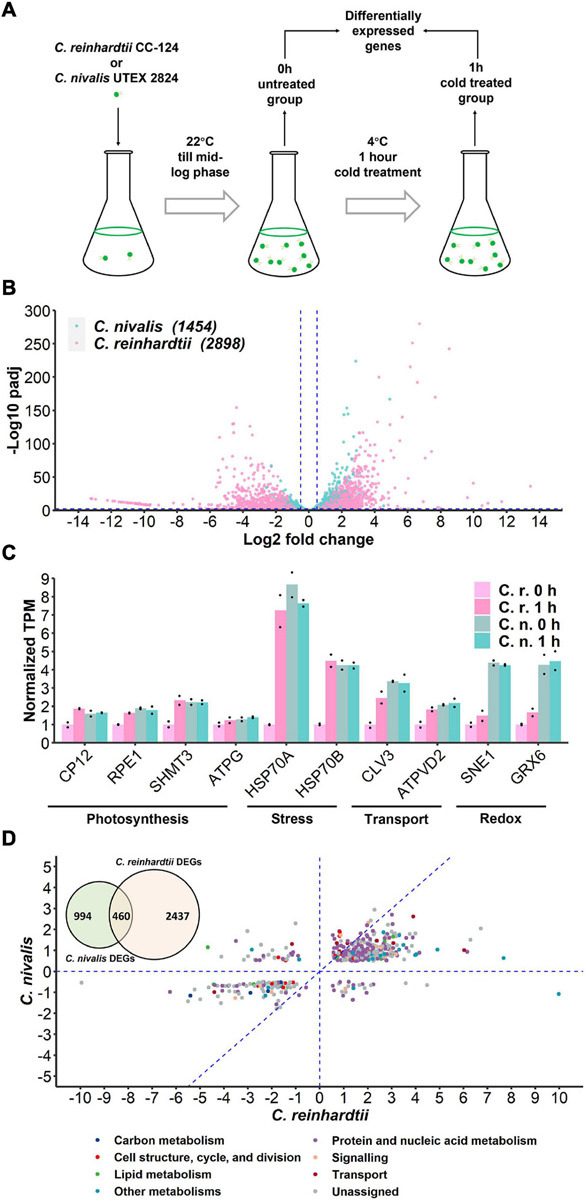
Transcriptome of *C. nivalis* is relatively stable compared to *C. reinhardtii*. **(A)** Schematic of sampling for RNA-seq. Algal cells were grown at 22°C until mid-log phase (0 h) and transferred to 4°C immediately for 1 h. Samples at 0 h and 1 h were collected for RNA-seq and differential gene expression analysis. **(B)** Volcano plot of DEGs in *C. nivalis* and *C. reinhardtii*. The blue dashed lines indicate the threshold (fold change > 2^0.5^, adjusted *P*-value < 0.01) for DEGs filtering. The number of DEGs is indicated in parentheses after the species name. padj, FDR-adjusted *P*-value. **(C)** Transcripts per million reads (TPM) of ten conserved genes. TPM values were normalized to TPM value of *C. reinhardtii* at 22°C. The dots indicate the two replicates for each bar. **(D)** Venn diagram in the upper-left corner shows overlapping DEGs in *C. nivalis* and *C. reinhardtii*. Scatter plot shows fold changes of the overlapping DEGs in the two species separately and the category of DEG is indicated by color. The blue dash lines (*x* = 0, *y* = 0, *x* = *y*) partition the DEGs to clarify the relationship between fold changes in *C. nivalis* and *C. reinhardtii*. LFC, log_2_ fold change.

To determine the differences in transcriptional changes in response to cold stress between *C. nivalis* and *C. reinhardtii*, we performed comparative transcriptomic analysis between *C. nivalis* UTEX 2824 and *C. reinhardtii* CC-124. Algal cells were cultured at 22°C until mid-log phase was reached. Half of the cells were harvested at 22°C, and the other half were harvested after 1 h of cold treatment (Figure 2A). We determined differentially expressed genes (DEGs) between the cold-treated group and the control group, and found that for *C. nivalis*, approximately 8.9% of 16,271 genes were differentially expressed (fold change > 2^0.5^, and adjusted *P*-value < 0.01). Of these DEGs, 853 genes were upregulated and 601 were downregulated. In *C. reinhardtii*, 14.8% of 19,525 genes were identified as differentially expressed with 1,580 upregulated and 1,318 downregulated (Supplementary Table 2). Among DEGs in *C. nivalis*, transcriptional regulators including Myb-like transcription factor (Contig21.98, log2 fold change [LFC] = 4.54), DEAD box RNA helicase (Contig8.58, LFC = 2.70), and bZIP transcription factor (Contig15.19, LFC = −1.72); nitrogen metabolism genes including nitrate transporter (Contig16.8, LFC = 2.84), and nitrate reductase (Contig16.6, LFC = 2.37); stress-related genes, including desiccation-related protein (Contig263.3, LFC = −2.23) were listed as the most differentially regulated genes (Table 1). To confirm transcriptional changes for the DEGs, we randomly selected 20 genes in *C. nivalis* and determined fold changes of the genes between 0 h and 1 h by quantitative RT-PCR. Through comparison of fold changes calculated from either RNA-seq or RT-PCR, there was an evident correlation (*R*^2^ = 0.736) between the results of the two methods (Supplementary Figure 2). For example, Contig16.6, Contig16.8, Contig99.34, Contig8.58, and Contig142.21 were upregulated by more than four-fold according to both RNA-seq and RT-PCR. These results confirm that the differential expression of these genes identified by the transcriptome analysis was reflected in the transcriptional response to the cold treatment.

**TABLE 1:** Top 20 most up or downregulated genes in *Chlamydomonas nivalis.*

**Gene ID**	**Description**	**LFC^a^**
**Upregulated in *C. nivalis***		
Contig51.26	–	4.91
Contig21.98	Myb-like transcription factor	4.54
Contig92.21	–	3.34
Contig206.23	–	3.07
Contig60.61	Transferase family	3.06
Contig1.372	WD repeat-containing protein	2.94
Contig43.36	–	2.89
Contig16.8	Nitrate transporter	2.84
Contig109.10	–	2.83
Contig6.214	–	2.71
Contig8.58	DEAD box RNA helicase	2.70
Contig142.21	CAP superfamily	2.69
Contig18.44	ABC transporter	2.61
Contig1.63	Adenine guanine permease	2.53
Contig6.215	–	2.49
Contig132.24	Cysteine-rich secretory protein	2.48
Contig52.87	Haloacid dehalogenase-like hydrolase	2.43
Contig9.105	Tetrapyrrole methylases	2.42
Contig16.6	Nitrate reductase	2.37
Contig84.65	Proline iminopeptidase	2.35
**Downregulated in *C. nivalis***		
Contig103.33	–	−2.86
Contig126.28	–	−2.58
Contig166.24	Ubiquitin carboxyl-terminal hydrolase	−2.34
Contig16.60	–	−2.29
Contig263.3	Desiccation-related protein	−2.23
Contig243.4	–	−2.14
Contig37.1	Leucine-rich repeat protein	−1.97
Contig169.11	AH/BAR domain superfamily	−1.87
Contig166.23	Guanylate cyclase	−1.86
Contig18.46	Transcription factor, zygote-specific	−1.85
Contig126.27	–	−1.85
Contig8.15	–	−1.79
Contig19.16	–	−1.72
Contig15.19	bZIP transcription factor	−1.72
Contig24.41	Phospholipid scramblase	−1.70
Contig69.15	–	−1.68
Contig35.3	–	−1.68
Contig16.51	–	−1.63
Contig91.17	–	−1.63
Contig61.14	Ankyrin repeat	−1.62

*^*a*^Log_2_ fold change.*

Fold change indicates the change of gene expression upon cold treatment. We firstly compared the variation in fold change of the genes to study differential expression between *C. nivalis* and *C. reinhardtii*. The adjusted *P*-value of DEGs was used to measure the significance of the fold change (Figure 2B). This analysis showed that the number of DEGs in *C. nivalis* was only 50.17% of that in *C. reinhardtii*, and that the fold changes of DEGs were no more than 2^5^ in *C. nivalis*, relative to the range of 2^10^ observed for *C. reinhardtii*. Approximately 15% of the DEGs in *C. reinhardtii* had fold changes greater than eight (log_2_ fold change ≥ 3), compared to only 0.3% of DEGs in *C. nivalis*. These results indicate that changes in gene expression were much more dramatic in *C. reinhardtii* than *C. nivalis*. Next, we quantified the absolute transcription level using transcripts per million reads (TPM). For all ten conserved genes related to photosynthesis, stress, transport, and redox categories that we selected, their transcription level was higher in *C. nivalis* than in *C. reinhardtii* at 22°C (Figure 2C). After transfer to 4°C, the transcription level of these genes increased in *C. reinhardtii* but remained stable in *C. nivalis*. This result suggests that some genes upregulated under cold stress in *C. reinhardtii* maintain high expression in *C. nivalis*, indicating that the expression levels of these genes at 22°C in *C. nivalis* were sufficient for their functions at low temperatures.

As they belong to the same genus, *C. nivalis* and *C. reinhardtii* share 11,022 homologous genes. However, only 460 homologous DEGs were identified in both species, which were 31.64% and 15.87% of total DEGs in *C. nivalis* and *C. reinhardtii*, respectively (Figure 2D, Venn diagram). The fold changes of the 460 intersected genes were compared between *C. nivalis* and *C. reinhardtii* (Figure 2D). Even among these homologous DEGs, 56 genes (12.7% of shared DEGs) were inversely regulated between *C. nivalis* and *C. reinhardtii* (located in the second or fourth quadrants of Figure 2D). These genes were DEGs belonging to transcription factors, chaperones, and flagellar proteins (Supplementary Table 3). For example, a C3H zinc finger transcription factor and two chaperones (ClpB and ClpD chaperone) were upregulated in *C. nivalis*, but downregulated in *C. reinhardtii*. Similarly, the expression of a DEAD box RNA helicase, a bZIP transcription factor, a Myb-like transcription factor, and a nuclear chaperone was decreased in *C. nivalis* but increased in *C. reinhardtii*. Moreover, 339 DEGs were uniquely detected in the *C. nivalis* genome (Supplementary Table 4). Together, these analyses indicate that transcriptional changes in *C. nivalis* is different compared to *C. reinhardtii* in response to cold.

### *De novo* Assembled Transcriptome Shows Stability in *C. nivalis*

**FIGURE 3:**
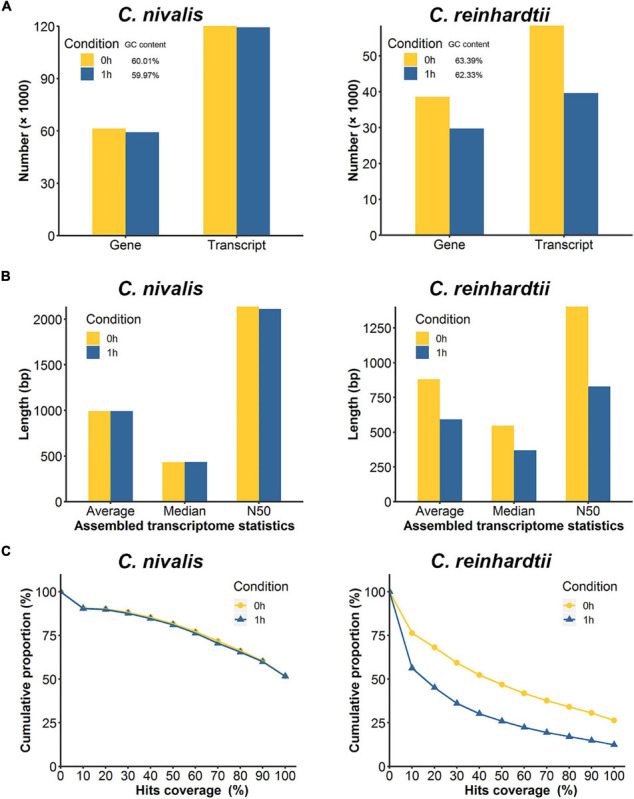
Comparison of *de novo* assembled transcripts between *C. nivalis* and *C. reinhardtii.* **(A)** Quantity of transcripts and corresponding genes in *de novo* assembled transcriptomes at 0 h and 1 h (percent GC content is indicated in the legend). **(B)** The length statistics of the transcriptomes at 0 h and 1 h. For each transcriptome the average, median, and N50 transcript lengths are provided. **(C)** For each transcriptome, we matched the transcripts to the reference genome, and determined the coverage of the reference gene length aligned to the transcripts, then plotted the cumulative proportions of total reference genes with certain coverage. The starting point of the trend line is set as hits coverage 0% and cumulative proportion 100%.

To further study the differences in transcriptome dynamics between the two species, the RNA-seq data were assembled into transcriptomes *de novo*. The transcript number, transcript length, and transcript integrity were compared between 0 h and 1 h. We observed that 120,108 transcripts corresponding to 61,356 genes (predicted “genes” and “transcripts” from the *de novo* assembled transcriptome) were assembled in the *C. nivalis* transcriptome at 22°C, while 119,385 transcripts matching 59,222 genes were assembled at 4°C, which indicated consistency between the temperatures (Figure 3A, left panel). At the two temperatures, the N50 (the sequence length of the shortest transcript at 50% of the total transcript length) and the average length of the transcripts remained the same (Figure 3B, left panel), indicating that the transcriptome did not change in *C. nivalis* in terms of transcript length when the temperature decreased. However, in *C. reinhardtii*, the number of transcripts decreased from 58,422 to 39,594 after cold stress (Figure 3A, right panel). Additionally, the N50 of *C. reinhardtii* transcripts decreased by 40.91%, from 1,403 bp to 829 bp, and the average length was shortened by 33% (Figure 3B, right panel). To quantify the integrity of transcripts, we matched the transcriptome to the reference database, examined the percentage of the best matched gene’s length being mapped to the transcripts, and plotted the cumulative proportion of genes by a certain coverage of total genes (Figure 3C). For example, in the *C. nivalis* database, 8,397 genes were found to be covered by a full-length transcript (100% on the *x*-axis) in the transcriptome of the 0 h group, which account for 51.6% of the total 16,271 genes, while 13,298 genes were covered by at least half length (50% on the *x*-axis), accounting for 81.7% of total genes. As shown in the graph, the trend lines of the 0 h and 1 h groups in *C. nivalis* almost coincided, indicating that the immediate cold did not influence the integrity of transcripts. In contrast, *C. reinhardtii* had 26.4% of total genes with 100% coverage in the transcriptome of the 0 h group, but only 12.4% in the 1 h group, indicating that *C. reinhardtii* cells might experience large-scale change of the transcriptome. In summary, this analysis suggests that *C. nivalis* better maintains homeostasis in the transcriptome in response to temperature fluctuations relative to *C. reinhardtii*.

### Transcription Factors and RNA-Binding Proteins as Regulatory Genes in Response to Cold in *C. nivalis*

**FIGURE 4:**
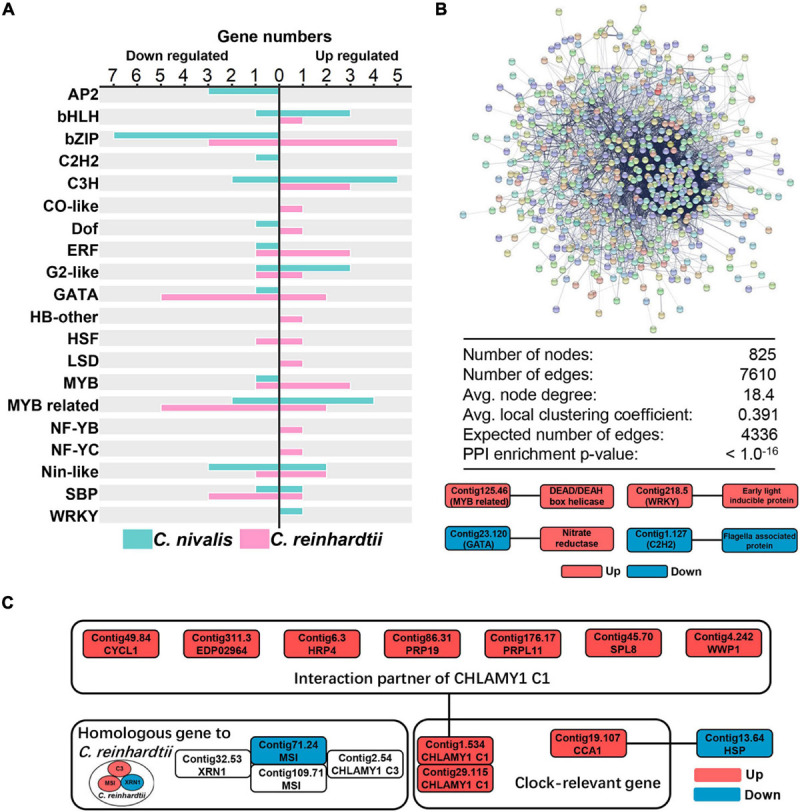
Candidate transcription factors and RNA-binding proteins participate in the cold response of *C. nivalis*. **(A)** Numbers of differentially expressed transcription factors in *C. nivalis* and *C. reinhardtii*, classified into transcription factor families. For each family, upregulated and downregulated genes are indicated by the direction of the axis. **(B)** Graph and table (top) showing protein–protein interaction analysis of DEGs in *C. nivalis* based on STRING database. DEGs are shown as colored circles and named as nodes (colors are used as a visual aid to identify the nodes), and interactions are shown as lines and named as edges. The graph below represents the interaction between differentially expressed transcription factors and genes related to metabolism in *C. nivalis*. Avg., average. PPI, protein–protein interaction. **(C)** Interaction network of differentially expressed clock-relevant genes in *C. nivalis*. Homologous genes differentially expressed in *C. reinhardtii* are also shown.

Transcriptome analysis showed that changes in gene expression were lower in *C. nivalis* than in *C. reinhardtii* during temperature decrease. We next sought to determine whether these differences were reflected in the regulatory genes of the transcriptome. As described previously, transcription factors were highly represented among the most up or downregulated genes in *C. nivalis* (Table 1). For further analysis, transcription factors in DEGs were also predicted using PlantTFDB, a database collecting plant transcription factors (Supplementary Table 5). We identified 19 upregulated and 25 downregulated transcription factor genes in *C. nivalis*. In contrast, *C. reinhardtii* had 30 upregulated and 21 downregulated transcription factors genes. The pattern of transcription factor expression was highly divergent between *C. nivalis* and *C. reinhardtii* (Figure 4A). Notably, most of the bZIP family transcription factors were downregulated in *C. nivalis* but upregulated in *C. reinhardtii* in response to cold. Some families were uniquely regulated, such as AP2, of which three transcription factors were downregulated in *C. nivalis*, but none in *C. reinhardtii*. This result indicates that although a similar number of transcription factors were differentially expressed in both algae, *C. nivalis* showed unique differential expression of specific transcription factors and families in response to cold.

To characterize the specific transcription factors involved in the regulatory network in *C. nivalis*, the STRING database was used to predict protein–protein interactions (PPI) between all DEGs. For *C. nivalis*, a total of 825 DEGs (nodes) comprised a network covering 7,610 protein–protein interactions (edges) (Figure 4B, upper panel). This number of DEGs with PPI was significantly greater than the expected number of interactions (4,336 edges; *P* < 10^–16^), indicating close interconnections among these DEGs in cold acclimation. Twelve transcription factors were found to have 34 interacting genes (Supplementary Table 6). Intriguingly, most of the transcription factors were upregulated or downregulated consistently with their interacting genes, especially for Contig22.73, an upregulated C3H domain containing transcription factors that had 19 upregulated interactors. Several genes encoding well-known proteins were characterized as interacting with transcription factors, including nitrate reductase, DEAD box RNA helicase, early light-inducible protein, and flagellar associated protein (Figure 4B, lower panel). Additionally, we identified a circadian clock-associated (CCA) gene Contig19.107. The gene encodes a Myb-related transcription factor, which interacted with the heat-shock protein Contig13.6.

In addition to transcription factors, the dynamics of the transcriptome are regulated by RNA-binding proteins (RBPs) (Hentze et al., 2018). Based on the STRING database, we characterized several RBPs that interacted with transcription factors, such as DEAD box RNA helicases and circadian rhythm-related genes. Using the PFAM domain search (offered by STRING), we identified helicase conserved C-terminal domain containing genes. Of these genes over 75% are DEAD box RNA helicases, which are reported to be cold stress regulators in *Arabidopsis thaliana* (Liu Y. et al., 2016). In total, 16 DEAD box RNA helicases were identified in DEGs, including the most upregulated genes Contig8.58 (LFC = 2.70), Contig46.21 (LFC = 2.26), and Contig8.52 (LFC = 2.21), indicating that the DEAD box RNA helicase family was essential for regulating the transcriptome of *C. nivalis*.

In the above analysis, we identified transcription factor Contig19.107 as a CCA gene, related to circadian rhythms. CHLAMY1 is a circadian RBP in *C. reinhardtii* (Iliev et al., 2006). CHLAMY1 C3 subunit interacts with two proteins, XRN1 and Musashi, which cooperate to confer thermal acclimation to *C. reinhardtii* (Li et al., 2018). In our work, CHLAMY1 C3 subunit, XRN1, and Musashi were all upregulated in *C. reinhardtii*. Comparatively, in *C. nivalis*, neither C3 nor XRN1 was DEGs, and only one transcript of Musashi (Contig71.24) was downregulated. However, there were two transcripts (Contig1.534 and Contig29.115) encoding the other subunit of CHLAMY1, the C1 subunit. These transcripts were both upregulated upon the temperature change in *C. nivalis* (Figure 4C). Furthermore, CHLAMY1 C1 was identified as interacting with seven DEGs, including cyclin, hydroxyproline-rich glycoprotein (HRP), and pre-mRNA-processing factor (PRP). Our results suggest that clock-relevant proteins such as CHLAMY1 C1 and CCA are key members in cold acclimation in *C. nivalis*, indicating *C. nivalis* may have different clock-relevant genes related to cold response compared to *C. reinhardtii*. Combined with the previous analysis on PPI, our findings indicate that *C. nivalis* possesses a robust regulatory network, containing transcription factors, DEAD box RNA helicases, and clock-relevant proteins. All the results above indicate that a portfolio of special transcription factors and RNA-binding proteins work together as regulatory genes in response to cold stress in *C. nivalis*.

### Differences in the Regulation of Biological Pathways Between *C. nivalis* and *C. reinhardtii*

**FIGURE 5:**
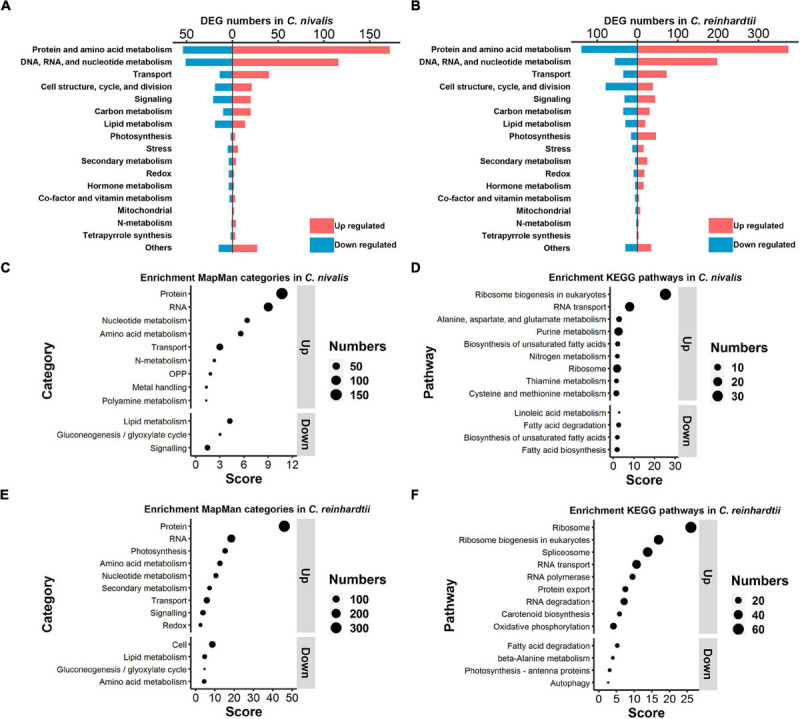
Functional classification and enrichment analysis of DEGs in *C. nivalis* and *C. reinhardtii.* **(A,B)** Numbers of *C. nivalis* DEGs **(A)** and *C. reinhardtii* DEGs **(B)** in MapMan categories. For each category, upregulated and downregulated genes are indicated by color. **(C–F)** The bubble plots of enriched MapMan categories **(C)** and enriched KEGG pathways **(D)** in *C. nivalis*; enriched MapMan categories **(E)** and enriched KEGG pathways **(F)** in *C. reinhardtii.* Only categories with scores > 1.3 are listed, separated as up or downregulated genes. Score for each category was calculated using the *P*-value (Score = –log_10_ [*P*-value]) in enrichment analysis. OPP, the oxidative pentose phosphate pathway.

To explore the differences in functional genes between *C. nivalis* and *C. reinhardtii*, we carried out classification and enrichment analysis of DEGs from both algae. In *C. nivalis*, DEGs were classified into 18 functional categories based on the MapMan database (Figure 5A). Notably, 27% of DEGs belonged to either protein and amino acid metabolism, or DNA, RNA, and nucleotide metabolism, suggesting that the processes of replication, transcription, and translation were significantly regulated during temperature decrease in *C. nivalis*. As for other important categories, 54 DEGs were related to cellular transport, 40 to cell structure and cell cycle proteins, 41 to signal transduction, and 63 to carbon and lipid metabolism proteins. Interestingly, only five photosynthesis related genes were differentially expressed, consistent with the stable efficiency displayed in photosystem II (Figure 1C). Additionally, several DEGs from biotic and abiotic stress response proteins, nitrogen metabolism, and hormone metabolism were identified as functional gene categories that altered in response to temperature change. The global category distribution of DEGs in *C. reinhardtii* was similar to that in *C. nivalis* (Figure 5B). For example, genes related to the metabolism of proteins, nucleic acids, amino acids, and nucleotides, overwhelmingly ranked as the most numerous in DEGs of both algae. The result reveals the conserved cold shock pathways shared by both algae. Transport and signaling pathways also made up a high proportion of the DEGs in both algae because these pathways are vital in turbulent environments. However, unlike *C. nivalis*, DEGs belonging to cell structure, cell cycle, and cell division were mostly downregulated in *C. reinhardtii*. Additionally, there were 62 photosynthesis-related DEGs with 46 upregulated and 16 downregulated in *C. reinhardtii*, much more than five photosynthesis-related DEGs in *C. nivalis*.

After the classification of DEGs we performed enrichment analysis based on the MapMan and KEGG database separately. For MapMan enrichment in *C. nivalis*, protein, RNA, nucleotide, amino acid metabolism, and transport gene categories ranked highly among the upregulated genes, which is consistent with the classification results (Figure 5C). For example, *N*-metabolism genes, including nitrate reductase and nitrite reductase, were found to be enriched in DEGs. Moreover, four oxidative pentose phosphate (OPP) pathway genes, including glucose-6-phosphate dehydrogenase (G6PDH) and 6-phosphogluconate dehydrogenase (PGD); three metal handling genes; and two polyamine metabolism genes, including spermine synthase and ornithine decarboxylase, were enriched in upregulated genes. On the contrary, lipid metabolism, gluconeogenesis/glyoxylate cycle, and signaling categories were enriched as key downregulated categories. Consistently, KEGG analysis showed that ribosome biogenesis, metabolism of nucleic acid (purine), and metabolism of amino acid (alanine, aspartate, glutamate, cysteine, and methionine) were upregulated in *C. nivalis* (Figure 5D). In addition, most of the enriched downregulated KEGG pathways belonged to lipid metabolism, suggesting that remodeling of lipid metabolism took place in response to cold. Similar to *C. nivalis*, we identified protein, RNA, nucleotide metabolism, amino acid metabolism, and transport appeared in up-regulated MapMan categories (Figure 5E); ribosome biogenesis in eukaryotes, and RNA transport in up-regulated KEGG pathways (Figure 5F). In contrast with *C. nivalis*, nitrogen metabolism and the OPP pathway were not enriched, while photosynthesis ranked third among the upregulated categories in *C. reinhardtii*. To study the differences of the regulated pathways between *C. reinhardtii* and *C. nivalis*, we further compared the number of DEGs classified into the MapMan subcategories (Supplementary Figure 3), and then used GO topology graphs to display GO enrichment analyses in the two species (Supplementary Figure 4). We observed that photosynthesis related genes were significantly upregulated in *C. reinhardtii*, including genes related to Calvin cycle, carbon concentration mechanism, light reaction, and photorespiration (Supplementary Figure 3E). The GO enrichment graph consistently indicated that DEGs were significantly enriched within the GO term “chloroplast” in *C. reinhardtii*, but not in *C. nivalis* (Supplementary Figure 4A). These results indicate that the transcriptional change in *C. nivalis* differs from *C. reinhardtii* in biological pathways such as photosynthesis, nitrogen metabolism, and the OPP pathway.

### DEGs in Nitrogen Metabolism and Central Carbon Metabolism

**FIGURE 6:**
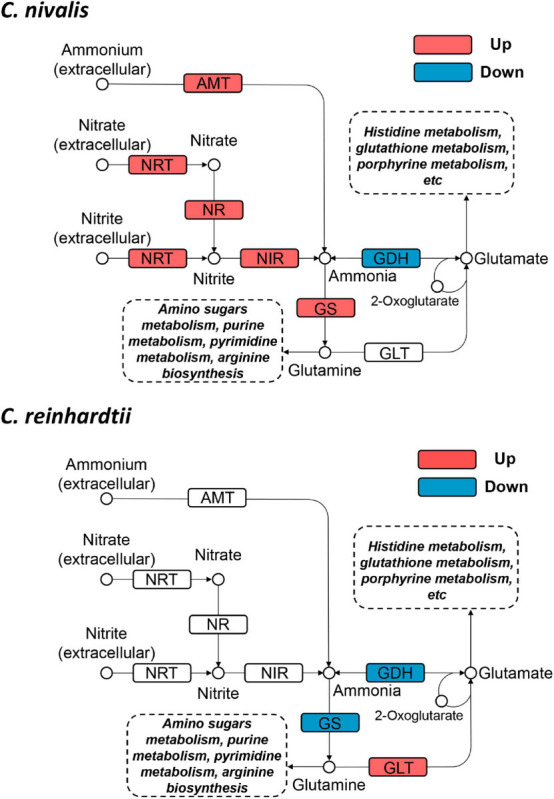
Differentially expressed genes in nitrogen metabolism in *C. nivalis* and *C. reinhardtii.* Arrows represent the direction of reactions. Enzymes are marked by solid rectangles, and substrates and products are marked by circles. Dashed rectangles indicate pathways connected to nitrogen metabolism. Upregulated and downregulated genes are indicated by red and blue, respectively. AMT, ammonium transporter; GDH, glutamate dehydrogenase; GLT, glutamate synthase; GS, glutamine synthetase; NIR, nitrite reductase; NR, nitrate reductase; NRT, nitrate/nitrite transporter.

To determine the functional genes important for cold response in *C. nivalis*, we focused on the metabolic pathway of N-metabolism, which was found to be enriched in DEGs in *C. nivalis* upon cold treatment (Figure 5C). Inorganic nitrogen compounds are assimilated by algae from extracellular environments in the form of nitrate and ammonium through nitrate and ammonium transporters, and reduced to nitrite and ammonia, which are then utilized in glutamine synthesis (Xu et al., 2012). As indicated by functional classification and enrichment analysis, nitrogen metabolism was characterized as an essential pathway for cold acclimation in *C. nivalis*. Based on the pathway map, nitrogen transporters, including five ammonium transporters (AMT) and one nitrate/nitrite transporter (NRT), one nitrate reductase (NR), and one nitrite reductase (NIR), were significantly upregulated (LFC > 2), suggesting that a large amount of extracellular ammonium, nitrate, and nitrite were taken up after cold shock, and that nitrate and nitrite may be further reduced to ammonia (Figure 6). In most algae, ammonia would then be transformed into glutamate or glutamine by glutamine synthetase (GS), which may serve as substrates for the synthesis of amino sugars, purine, pyrimidine, and arginine. However, in *C. reinhardtii*, no such regulation was detected, suggesting a unique regulation of nitrogen metabolism in *C. nivalis*.

**FIGURE 7:**
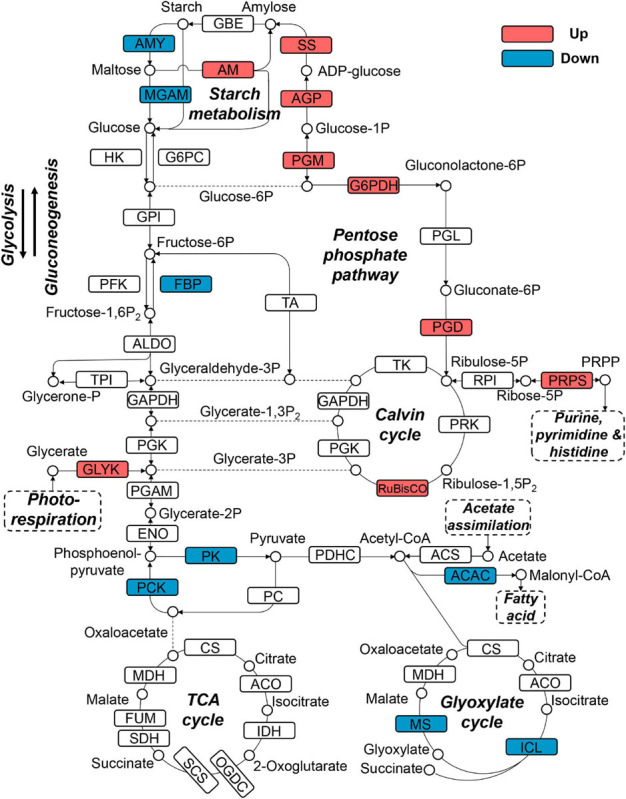
Differentially expressed genes in central carbon metabolism in *C. nivalis.* Arrows represent the direction of reactions. Enzymes are marked by solid rectangles, and substrates and products are marked by circles. Dashed rectangles indicate pathways connected to carbon metabolism. Upregulated and downregulated genes are indicated by red and blue, respectively. ACAC, acetyl-CoA carboxylase/biotin carboxylase; ACO, aconitate hydratase; AGP: glucose-1-phosphate adenylyltransferase; ALDO, fructose-bisphosphate aldolase; AM, amylomaltase (4-alpha-glucanotransferase); AMY, beta-amylase; CS, citrate synthase; ENO, enolase; FBP, fructose-1,6-bisphosphatase; FUM, fumarate hydratase; G6PC, glucose-6-phosphatase; G6PDH, glucose-6-phosphate dehydrogenase; GLYK, glycerate 3-kinase; GPI, glucose-6-phosphate isomerase; HK, hexokinase; ICL, isocitrate lyase; IDH, isocitrate dehydrogenase; MDH, malate dehydrogenase; MGAM, maltase-glucoamylase; MS, malate synthase; OGDC, 2-oxoglutarate dehydrogenase complex; PC, pyruvate carboxylase; PCK, phosphoenolpyruvate carboxykinase; PDHC, pyruvate dehydrogenase complex; PFK, phosphofructokinase; PGAM, phosphoglycerate mutase; PGD, 6-phosphogluconate dehydrogenase; PGK, phosphoglycerate kinase; PGL, 6-phosphogluconolactonase; PGM, phosphoglucomutase; PK, pyruvate kinase; PRK, phosphoribulokinase; PRPP, phosphoribosylpyrophosphate; PRPS, ribose-phosphate pyrophosphokinase (PRPP synthase); RPI, ribose 5-phosphate isomerase; RuBisCO, ribulose-bisphosphate carboxylase; SCS, succinyl-CoA synthetase; SDH, succinate dehydrogenase; SS, starch synthase; TA, transaldolase; TK, transketolase; TPI, triosephosphate isomerase.

**FIGURE 8:**
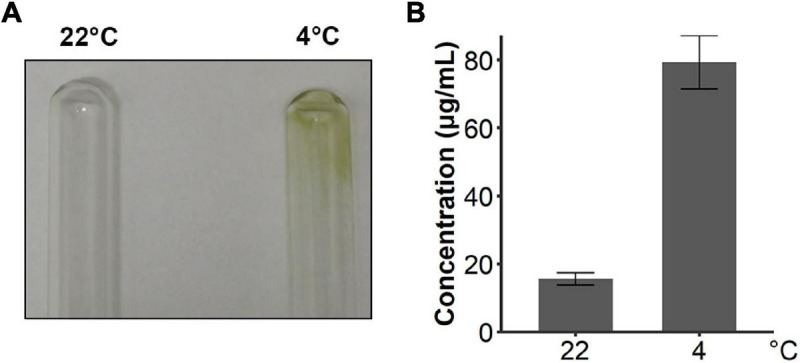
*C. nivalis* secreted more EPS in cold environments. **(A)** Photograph of two tubes where the walls were adhered by cultures of *C. nivalis* growing at 22°C and 4°C separately. **(B)** Bar plot shows concentrations of EPS in *C. nivalis* growing at 22°C and 4°C for 3 days. Error bars represent the standard deviations of three biological replicates.

Since snow algae participate in carbon cycling in the snowfield, we then investigated functional genes in central carbon metabolism in *C. nivalis*. The central carbon metabolic pathway map includes glycolysis/gluconeogenesis, pentose phosphate pathway, Calvin cycle, TCA cycle, glyoxylate cycle, and starch metabolism (Figure 7). As indicated by enrichment analysis (Figure 5C), five DEGs involving the oxidative pentose phosphate (OPP) pathway were characterized. Glucose-6-phosphate dehydrogenase (G6PDH) and 6-phosphogluconate dehydrogenase (PGD) are rate-limiting enzymes in the OPP pathway and were both upregulated DEGs in *C. nivalis*, indicating that the pathway was highly activated after cold shock. Meanwhile, glucose-1-phosphate adenylyltransferase (AGP) and starch synthase (SS), which convert glucose-1P into amylose and starch, were also upregulated, but beta-amylase (AMY) and maltase-glucoamylase (MGAM), which convert starch into glucose, were downregulated. The result suggests that cold stress induced the transformation of monosaccharides into polysaccharides in *C. nivalis.* The final product of carbon flow might be exopolysaccharides (EPS), which is produced by psychrophiles under cold conditions (De Maayer et al., 2014). We noticed that more *C. nivalis* cells adhered to the glass wall when cultured at 4°C than the cells grown at 22°C (Figure 8A). To confirm this, the EPS of *C. nivalis* were extracted, and the EPS content at 4°C was determined to be nearly four times that at 22°C (Figure 8B), suggesting that a large number of polysaccharides were synthesized, some of which accumulated as EPS, leading to increased adhesion and cold resistance.

Upregulation of genes coding for RuBisCO and phosphoribosylpyrophosphate synthase (PRPS) was observed in *C. nivalis* as well. Ribulose 5-phosphate (Ribulose-5P) produced from the pentose phosphate pathway can be utilized as the precursor of ribulose 1,5-bisphosphate (RuBP) in the Calvin cycle. It can also be catalyzed by PRPS for purine metabolism, which is a critical pathway according to KEGG enrichment (Figure 5D). Nevertheless, isocitrate lyase (ICL) and malate synthase (MS) in the glyoxylate cycle were downregulated in *C. nivalis* (Figure 7). The glyoxylate cycle usually works as a bypass to the TCA cycle, and helps to convert C-2 carbon, such as acetyl-CoA, generated from lipid degradation, to glucose. Therefore, reduction in the glyoxylate cycle suggests that utilization of fatty acids as carbon sources for gluconeogenesis is slowed at lower temperatures in *C. nivalis*.

In *C. reinhardtii*, enzymes in the Calvin cycle, such as RuBisCO, transketolase, phosphoribulokinase, and phosphoglycerate kinase were upregulated (Supplementary Figure 5). Besides ICL and MS, which were downregulated in *C. nivalis*, enzymes including isocitrate dehydrogenase (IDH) in the TCA cycle, malate dehydrogenase, and citrate synthase in the TCA and glyoxylate cycles, were also downregulated in *C. reinhardtii*. It was inferred that the Calvin cycle was increased, and the TCA cycle was decreased in response to cold in *C. reinhardtii*, but this regulation was not observed in *C. nivalis*. All the results above indicate that nitrogen metabolism, the OPP pathway, and polysaccharide synthesis are important biological pathways in response to temperature decrease in *C. nivalis*.

### HGT in *C. nivalis*

In addition to analyzing genes homology to *C. reinhardtii*, we explored functional genes which uniquely exist in *C. nivalis* genome. Among 339 DEGs uniquely detected in *C. nivalis*, we identified nine annotated genes that were best matched to prokaryotic and fungal species, and one IBP encoding gene, which may be candidate horizontally transferred genes (Supplementary Table 4). Three of the ten candidates were certified based on construction of phylogenetic trees, including the IBP encoding gene Contig23.48, glycosyltransferase encoding gene Contig7.197 and *S*-adenosyl-l-methionine (SAM) dependent carboxyl methyltransferase encoding gene Contig69.73 (Supplementary Figure 6). The upregulated gene Contig23.48 best matched IBP9 from the snow algae *Chloromonas brevispina*, which is reported to be acquired through HGT (Raymond, 2014). Unlike *Chloromonas brevispina*, which has 11 IBPs in its genome, we identified only two IBPs, Contig23.47 and Contig23.48 in *C. nivalis*. Expression of Contig23.48 increased four-fold during temperature fluctuations, a relatively large fold change in *C. nivalis* (rank 36th in 853 upregulated genes), indicating a significant role for IBP in cold response.

## Discussion

**FIGURE 9:**
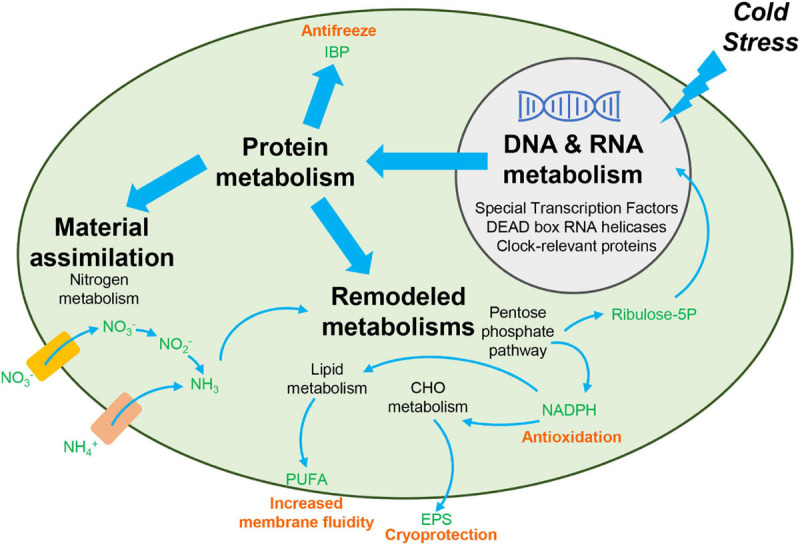
Overview of cold adaptation mechanisms in *C. nivalis.* Black bold text indicates the main processes in the cold adaptation mechanism, and black non-bolded text indicates the detailed pathways and genes related to the process identified in this study. Green text indicates the chemicals in the pathways. Pink text indicates the function of the compounds in cold resistance. Rectangles indicate the nitrate and ammonium transporters. Upon cold stress from the extracellular environment, signals upstream are transferred to regulate the processing of RNA and synthesis of functional proteins. The functional proteins then allow for increased material assimilation and remodeling of metabolisms, to produce specific chemicals needed for cold resistance. CHO, carbohydrate. EPS, exopolysaccharides. IBP, ice-binding protein. PUFA, polyunsaturated fatty acid. Ribulose-5P, ribulose 5-phosphate.

In this study, we employed transcriptomic analyses to explore cold adaptation mechanisms during temperature fluctuations in *C. nivalis*. To reveal the unique mechanisms that *C. nivalis* has evolved through survival in cold environments, we used the model organism *Chlamydomonas reinhardtii* for comparison, to distinguish the characteristics of psychrophiles and mesophiles. We observed that *C. nivalis* exhibited fewer changes in the transcriptome upon cold stress relative to *C. reinhardtii*, and the expression level of photosynthesis-related genes was almost constant in *C. nivalis* compared to *C. reinhardtii*. We then investigated the unique DEGs in *C. nivalis* including regulatory and functional genes. For the upstream regulatory network, specific transcription factors and RBPs were revealed as key regulators in *C. nivalis*. We also found that both species were characterized with a significant regulation of genes responsible for replication, transition, and expression of genetic information. For biological pathways downstream, nitrogen metabolism and the OPP pathway are uniquely regulated in *C. nivalis*. Besides genes homologous to *C. reinhardtii*, we identified one IBP encoding gene upregulated, which only exist in the *C. nivalis* genome. Based on these findings, we uncovered the cold adaptation mechanisms under temperature change in a typical psychrotolerant green alga at the transcriptomic level (Figure 9). In response to sudden cold stress, signals transfer through transcription factors and RBPs, including DEAD box RNA helicases and clock-relevant proteins, causing downstream regulation of the expression of functional enzymes. For instance, nitrogen assimilation increases to supply materials for remodeled metabolisms such as the pentose phosphate pathway, and the produced reducing power and intermediates are utilized in the synthesis of compounds such as lipids and polysaccharides, collaborating with IBPs to counteract the negative effects of cold stress.

### Relatively Stable Transcriptome of *C. nivalis*

Compared to the mesophilic alga *C. reinhardtii*, the transcriptome of *C. nivalis* was observed to change less during temperature fluctuations in two ways. First, the number of DEGs in *C. nivalis* was only half that of *C. reinhardtii*, and these DEGs had a smaller range of fold changes. Second, the *de novo* assembled transcriptome of *C. nivalis* remained consistent under sudden cold stress in terms of transcript number, transcript length, and transcript integrity, indicating fewer transcriptomic changes compared to *C. reinhardtii*. Transcriptomic analysis has been applied to the ice alga *C.* ICE-L to study acclimation to cold environments (Liu C. et al., 2016). Recently, in *C. reinhardtii*, gene expression in early response to cold was discussed using RNA-seq technology (Li et al., 2020). However, no comparative transcriptomic analysis between snow algae and *C. reinhardtii* has been conducted. In our work, we integrated the snow alga *C. nivalis* and mesophilic alga *C. reinhardtii* to investigate cold adaptation mechanisms for the first time, discovering the transcriptomic homeostasis during temperature changes in snow algae.

In the Antarctic alga strain *Chlorella vulgaris* NJ-7, nitrate reductases and cryoprotective proteins were found to be highly abundant to compensate their low activities in the cold environments (Wang et al., 2020). We observed that several functional genes were upregulated only in *C. reinhardtii*, while the constant expression level of the genes in *C. nivalis* was as high as the increased level in *C. reinhardtii* at 4°C. It is likely that the high abundance of these genes eliminates the need for temporary upregulation during temperature fluctuations, and leads to a stable transcriptome as in NJ-7. It is also possible that RNA processing may be impaired in *C. reinhardtii* but not in *C. nivalis* under cold stress, due to the significant change in the *de novo* assembled transcriptome of *C. reinhardtii*.

### Regulation Network in *C. nivalis* in Response to Temperature Change

The regulatory network in *C. nivalis*, which is likely made up of transcription factors, must be highly efficient to adapt to temperature changes while minimizing transcriptomic changes. When we compared the distribution of differentially expressed transcription factors into families between *C. reinhardtii* and *C. nivalis*, we found them to be highly divergent in expression patterns from each other. A recent study in *C. reinhardtii* stated that the transcription factors responsive to cold mostly belonged to the bZIP- and Myb-related families, consistent with our results (Li et al., 2020). Nonetheless, we also found differences between our result and theirs, such as the absolute number of transcription factors in each family, which likely arose from variance between experimental batches including algae strain, culture condition, sequencing, and data processing. To fully determine the function of the transcription factors in cold response, genetic manipulation in *C. reinhardtii* is required.

DEAD box proteins, which constitute the majority of RNA helicases, are involved in nearly all RNA-associated events, especially ribosome biogenesis, and are known to be important regulators of stress response (Cordin et al., 2006). In our study, 14 DEAD box RNA helicases were upregulated in *C. nivalis* in response to temperature changes. A previous study in *Arabidopsis thaliana* discovered the essential role of DEAD box RNA helicases in biotic and abiotic stress responses (Gong et al., 2005). For instance, the helicase family member AtRH7 interacts with a cold shock protein to protect plant growth under cold conditions (Liu Y. et al., 2016). Genomic and transcriptomic research inferred that DEAD box RNA helicases are also involved in cold acclimation in a psychrophilic archaeon (Chen et al., 2012b). Besides plants and archaea, there were 39 DEAD box RNA helicases identified in the transcriptome of the ice alga *C.* ICE-L, and several of them were upregulated upon cold treatment (Liu C. et al., 2016). Intriguingly, nine of the ten *C. nivalis* DEGs homologous to *C.* ICE-L belonged to DEAD box RNA helicases (Supplementary Table 7), and there were also 24 DEAD box RNA helicases found to be up regulated in *C. reinhardtii* (Supplementary Table 2). All the above suggests that the helicase family probably represents a conserved cold stress response in the *Chlamydomonas* genus. RNA helicases often function upstream of the gene regulatory network. Based on interaction analysis, we found that the transcription factor Contig125.46 interacted with a DEAD box RNA helicase Contig12.138, indicating a regulatory cascade from DEAD box RNA helicases to metabolic genes.

We also identified the transcription factor, Myb-related family member Contig19.107, as a CCA gene. A previous study inferred that the CCA gene functions in the phytochrome signal transduction pathway in *Arabidopsis thaliana* (Wang et al., 1997). In *C. reinhardtii*, the clock-relevant RBP CHLAMY1, consisting of the C1 and C3 subunits, was involved in a temperature-controlled network (Iliev et al., 2006; Voytsekh et al., 2008). Another relevant clock protein, XRN1, a 5′–3′ exoribonuclease, interacts with the CHLAMY1 subunits (Dathe et al., 2012). According to a recent study, the C3 subunit, XRN1, and Musashi interact with each other and cooperate to confer thermal acclimation to *C. reinhardtii* (Li et al., 2018). In our analysis, the C3 subunit, XRN1, and Musashi were identified as DEGs in *C. reinhardtii* consistently with previous studies, but were not differentially regulated in *C. nivalis*. Instead we found that CHLAMY1 C1 subunit, was upregulated and interacted with seven DEGs in *C. nivalis*, indicating that the C1 subunit plays an important role in the regulatory network. Thus, it is likely that while clock-relevant proteins in *C. nivalis* participate in the regulatory network, similar to *A. thaliana* and *C. reinhardtii*, the specific genes involved in the regulation are different.

### The Biological Pathways Related to Cold Adaptation in *C. nivalis*

According to the classification and enrichment of DEGs, one of the most distinguishing characteristics between *C. nivalis* and *C. reinhardtii* was related to the expression of genes in the photosynthesis pathway, which is indispensable to the survival of green algae. Only five genes belonging to the photosynthesis category were DEGs in *C. nivalis*, including the RuBisCO small subunit, glycerate kinase, and photosystem I reaction center subunit III. However, 64 DEGs were identified in the photosynthesis category for *C. reinhardtii* and these could be further classified into Calvin cycle, carbon concentrating mechanism (CCM), light reaction, and photorespiration. A proteomic analysis in *C. reinhardtii* showed similar results, in which light-harvesting complex proteins, enzymes belonging to the CO_2_ fixation pathway, and CCM were regulated in cold stress adaptation (Valledor et al., 2013). Another transcriptomic study also declared that genes related to the chloroplast apparatus changed after cold treatment in *C. reinhardtii* (Li et al., 2020). Yet, we discovered that snow algae exhibit stable expression of photosynthesis related genes in response to cold stress compared to mesophilic alga *C. reinhardtii*. Given these findings and that *C. nivalis* maintained the photosynthetic capacity better than *C. reinhardtii*, we conclude that the expression pattern of the photosynthesis pathway in *C. nivalis* at normal temperature was partially adaptative to cold, thus avoiding energy consumption of pathway remodeling during temperature fluctuations.

Nitrogen assimilation and the OPP pathway were revealed as two important pathways in response to temperature changes in *C. nivalis*, compared with *C. reinhardtii*. Nitrogen metabolism is regulated during cold acclimation, due to the impediment of nitrogen transport by cold stress and the demand for energy balance (Morgan-Kiss et al., 2006). High activity and upregulation of nitrate reductase has been observed in several psychrophilic algae (Di Martino Rigano et al., 2006; Chen et al., 2012a; Liu C. et al., 2016). We first clarified that the increase in nitrogen assimilation was a unique cold-adaptation mechanism in *C. nivalis* but not in *C. reinhardtii*. This is achieved through the upregulation of ammonium transporters, nitrate/nitrite transporters and reductases in *C. nivalis.* Although there are more than 20 transporters of nitrate, nitrite or ammonium in *C. reinhardtii*, none of them were identified as upregulated genes in our study. The increase in nitrogen assimilation probably indicated that the transcriptional level elevated in *C. nivalis* to meet the demand of more nitrogen compounds at low temperatures. Another possible cause of the upregulated assimilation is to compensate for the inefficiency of nitrogen transport under cold stress, as ammonium transporters and permeases in *C. reinhardtii* were reported to be increased by as much as eight-fold under nitrogen starvation (Lopez Garcia de Lomana et al., 2015). Moreover, we identified five ammonium transporters that were differentially expressed, indicating that besides nitrate/nitrite transporters and reductases, assimilation of extracellular ammonium ions was likely to be increased in *C. nivalis*, rendering diverse sources of nitrogen.

The pentose phosphate pathway involves oxidation of glucose, produces precursors for nucleotide and amino acid biosynthesis, and provides reducing power for anabolism and antioxidation (Stincone et al., 2015). A previous study in creeping bentgrass (*Agrostis stolonifera*) reported the role of the proline-associated pentose phosphate pathway in cold stress tolerance (Sarkar et al., 2009). In the arctic alga *Chlorella*-Arc, the pentose phosphate pathway has been found to play a role in response to thermal/cold stress (Song et al., 2020). We suggest that in snow algae, increasing the OPP pathway helped to supply ribose 5-phosphate and NADPH for biosynthesis reactions to resist cold stress. In general, carbon and nitrogen metabolism is regulated in alignment with the improved nitrogen assimilation and pentose phosphate pathway to maintain homeostasis at low temperatures, while the demand for cryoprotectants and antioxidants is satisfied in *C. nivalis*.

Lipid metabolism plays a pivotal role in cold response, especially the desaturation of fatty acids which help to maintain cell membrane fluidity at low temperatures. In *C. nivalis*, lipid regulation is considered an important adaptation to environmental changes such as salt stress, nitrate deprivation, and phosphate deprivation (Lu et al., 2012a,b, 2013). Meanwhile, specific unsaturated fatty acids including C16:2 and C18:2 were identified in the lipid profiling in cold-stressed *C. reinhardtii* (Valledor et al., 2013). In our transcriptomic analysis, we found the changes of lipid metabolism in *C. reinhardtii* and *C. nivalis* were similar (Supplementary Figure 4D). The expression of four fatty acid desaturases increased in *C. reinhardtii*, including linoleate desaturase, delta-9 desaturase, omega-3 desaturase, and acyl-ACP (acyl carrier protein) desaturase. The expression of all these desaturases increased in *C. nivalis*, as well as two omega-6 desaturases. Furthermore, the expression of fatty acid degradation related genes, including triacylglycerol lipases and acyl-CoA dehydrogenases, were downregulated in both algae, implying that a dramatic alteration of lipid composition occurs during cold acclimation. However, we observed downregulation of four genes related to glycerol metabolism in *C. reinhardtii*, including glycerol kinase and glycerol-3-phosphate dehydrogenases, but the FAD-dependent glycerol-3-phosphate dehydrogenase was upregulated two-fold in *C. nivalis*. All these data suggest that lipid metabolism regulation functions as a conserved mechanism during cold stress in the *Chlamydomonas* genus.

### Horizontally Transferred Genes Are Identified in *C. nivalis* DEGs

Genome sequencing in the last decade has revealed HGT as a major force that continuously reshapes genomes throughout evolution (Daubin and Szollosi, 2016). In the DEGs of *C. nivalis*, one SAM dependent carboxyl methyltransferase and one glycosyltransferase were certified to be acquired through HGT. We also identified a DEG encoding IBP similar to IBP9 in *Chloromonas brevispina*, which might have the same origin from bacteria. IBP protects cells by inhibiting ice crystal nucleation. In *Chloromonas brevispina*, 11 IBPs shared a large domain called DUF3494 and were proven to be horizontally transferred genes (Raymond, 2014). IBPs originating from HGT were also discussed in the transcriptomic and genomic study of *C.* ICE-L (Liu C. et al., 2016; Zhang et al., 2020), and in the fungus *Antarctica psychrotrophicus* (Arai et al., 2019). In addition to IBPs, heat-shock proteins in *C.* ICE-L, and ATPases in the red alga *Galdieria sulphuraria* were identified as horizontally transferred genes that also play a role in cold acclimation (Liu et al., 2018; Rossoni et al., 2019). SAM dependent carboxyl methyltransferases catalyze the transfer of a methyl group from SAM to a variety of substrates, indicating specific methylated derivatives in *C. nivalis*. Glycosyltransferases catalyze glycosyl transfer to synthesize a carbohydrate, glycoside, oligosaccharide, or polysaccharide. The horizontally transferred glycosyltransferases might lead to specific forms of glycosidic linkages and heterogeneous sugar molecules, and were regulated to fit for the changed environment. Moreover, glycosyltransferase Contig7.197 was found to match proteins in *Methylobacter tundripaludum*, a psychrotolerant aerobic methane-oxidizing bacterium, providing *C. nivalis* with specific capacities of modifying polysaccharides for cold adaptation. Heterogeneous expression of these genes in *C. reinhardtii* may be a feasible way to further investigate their function.

In summary, the snow alga *C. nivalis* has evolved a high-efficiency transcriptional regulation network capable of remodeling metabolism for cold tolerance, and benefits from horizontally transferred genes. Owing to these advantages, *C. nivalis* can adapt to temperature fluctuations, survive, and become a major producer in low-temperature ecosystems.

## Data Availability Statement

The datasets presented in this study can be found in online repositories. The names of the repository/repositories and accession number(s) can be found below: https://www.ncbi.nlm.nih.gov/, PRJNA659384.

## Author Contributions

KH, ZP, and GL contributed to the experiment design. ZP performed the experiment work, analyses, and wrote the manuscript. KH and GL revised the manuscript. All authors contributed to the article and approved the submitted version.

## Conflict of Interest

The authors declare that the research was conducted in the absence of any commercial or financial relationships that could be construed as a potential conflict of interest.

## Footnotes

1 https://phytozome.jgi.doe.gov

2 ftp://ftp.ncbi.nih.gov/blast/db/
