# Supplementary material for: Cold Adaptation Mechanisms of a Snow Alga Chlamydomonas nivalis During Temperature Fluctuations
Source: Front Microbiol. 2021 Jan 11;11:611080. doi: 10.3389/fmicb.2020.611080 (PMC7874021; doi:10.3389/fmicb.2020.611080)
Supplement: Supplementary file 1 [file Data_Sheet_1.docx]

Supplementary Material

# Supplementary Figures


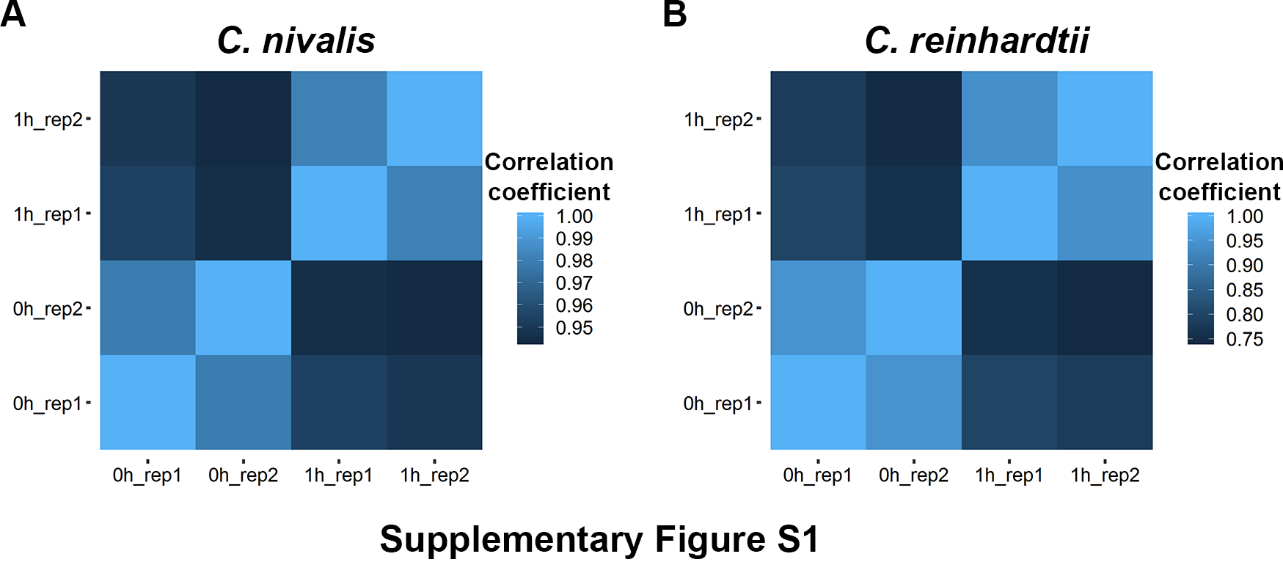


# Supplementary Figure S1. Heat maps showing correlation coefficients between RNA-seq samples based on gene count matrices in *C. nivalis* and *C. reinhardtii*.


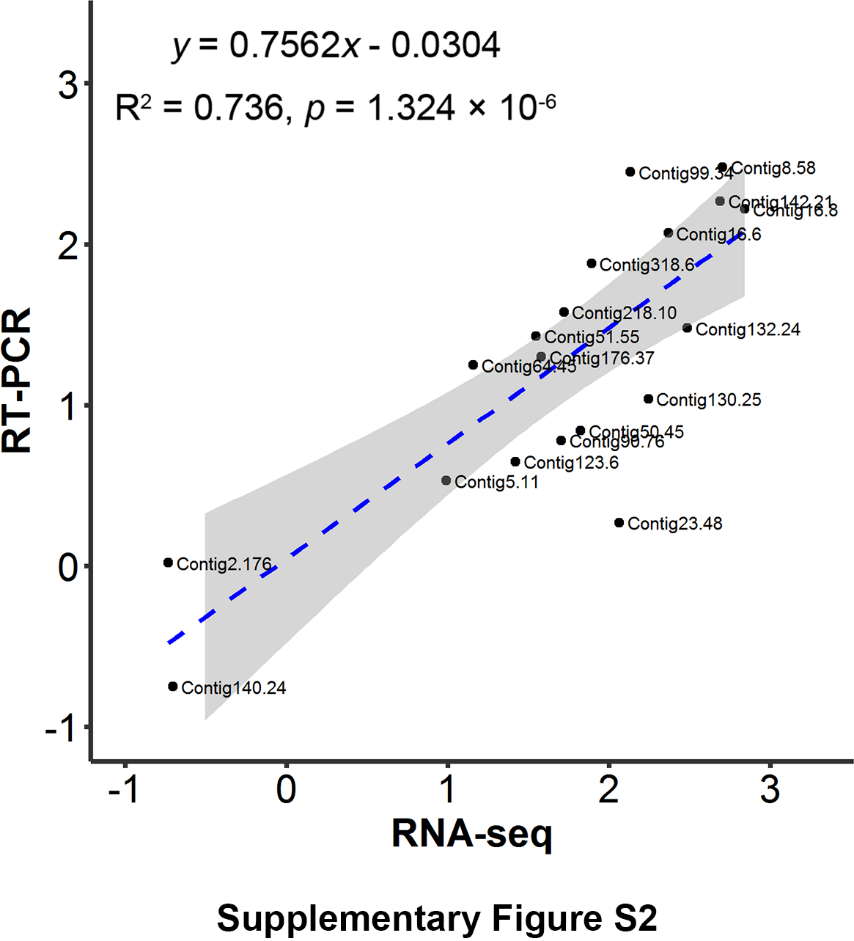


# Supplementary Figure S2. Correlative analysis of fold changes resulting from RNA-seq and RT-PCR

Scatterplot comparing fold changes between RNA-seq and RT-PCR. The blue dashed line indicates the linear regression line. The gray area indicates the 95% confidence interval. The linear regression formula, R^2^ (coefficient of determination), and *P*-value are shown in the upper-left corner. The numbers on the x and y-axes indicate log_2_ fold change of the DEGs.


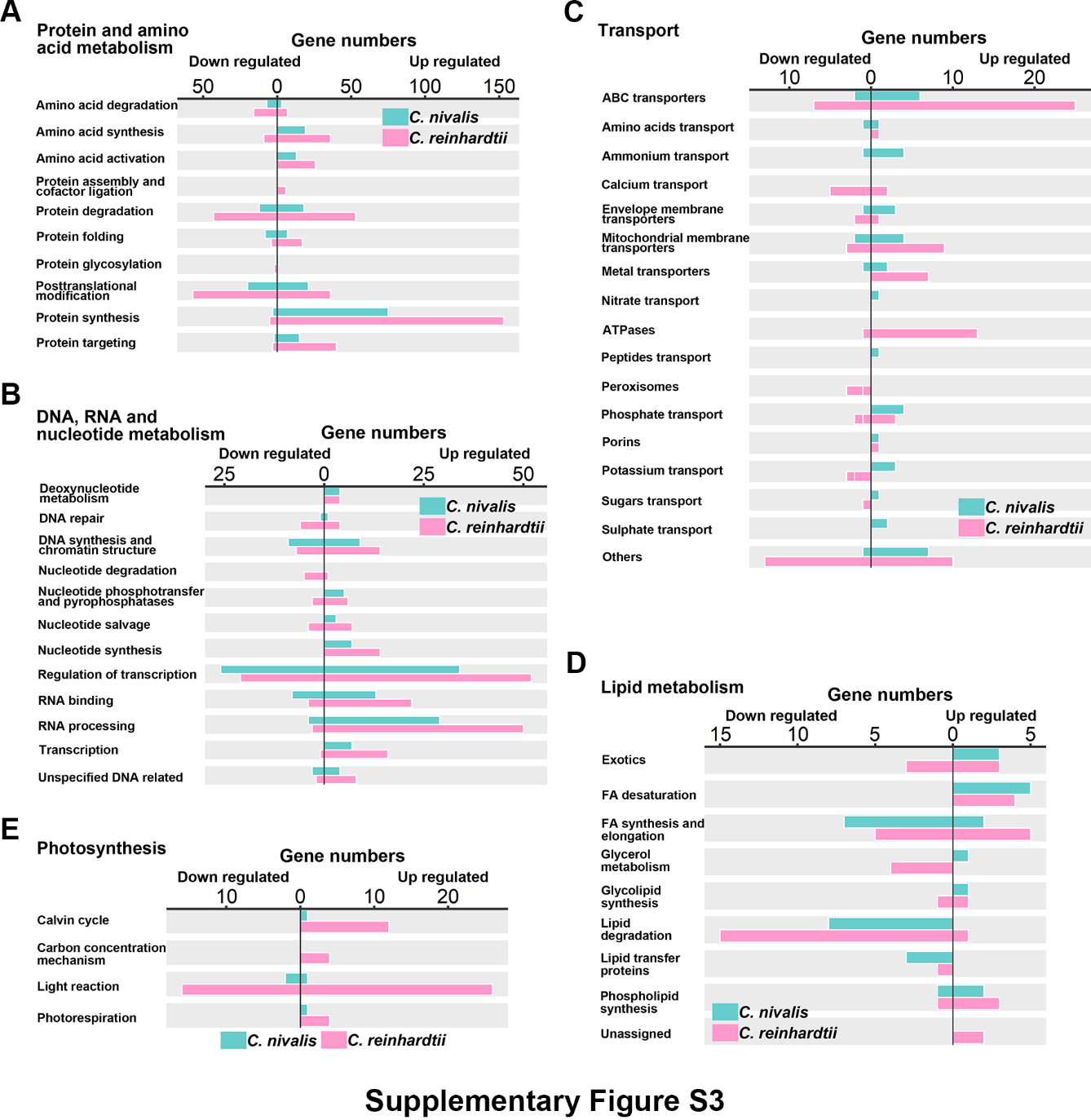


# Supplementary Figure S3. Functional classification of DEGs in *C. nivalis* and *C. reinhardtii* based on the MapMan database.

For further analysis, DEGs from protein and amino acid metabolism category (A), DNA, RNA, and nucleotide metabolism category (B), transport category (C), lipid metabolism category (E), and photosynthesis category (F) were classified into subcategories. For each subcategory, DEGs from *C. nivalis* and *C. reinhardtii* are indicated by blue and red, up and downregulated genes are indicated by the direction of the x-axis, respectively.


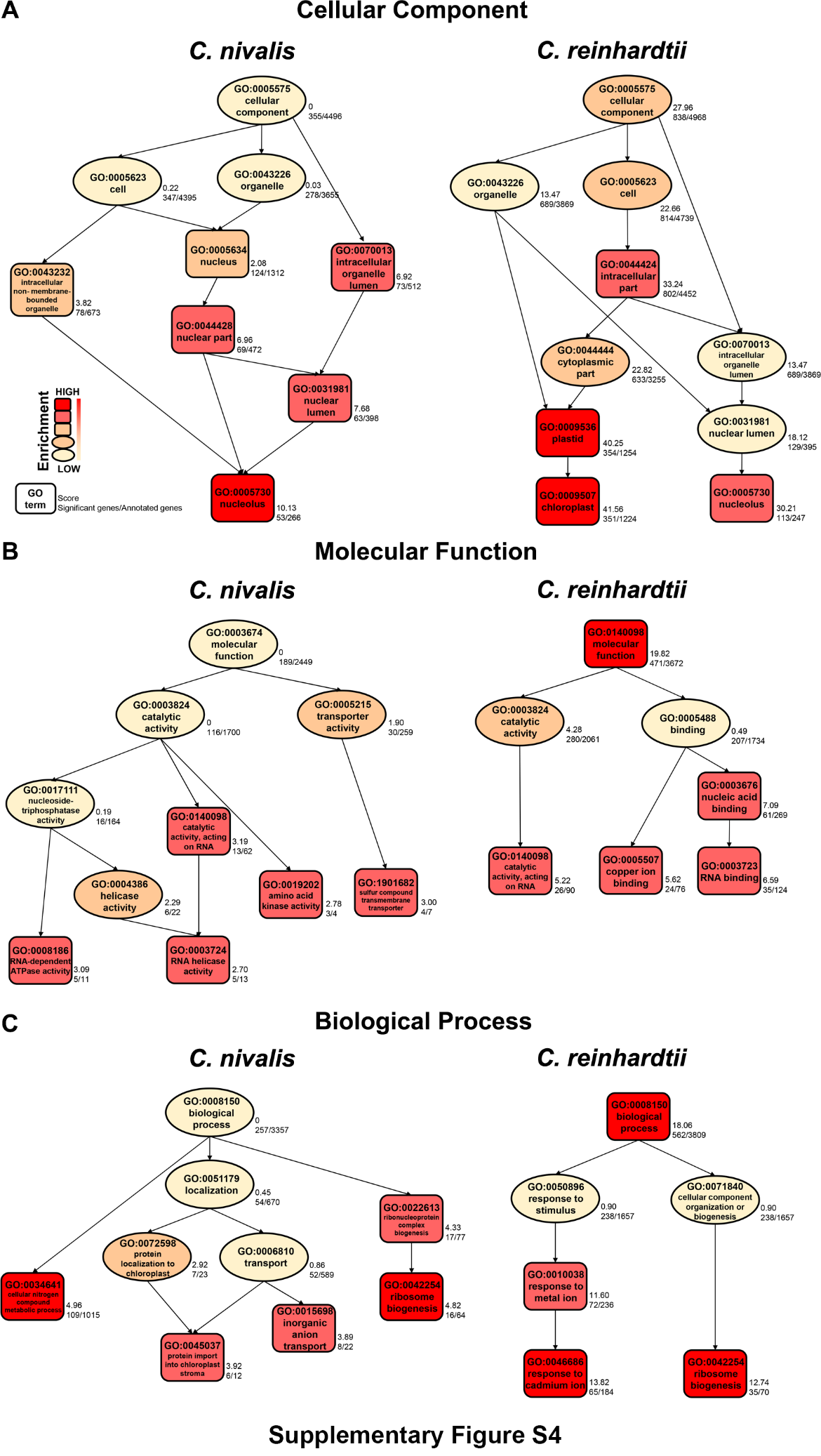


# Supplementary Figure S4. Enrichment analysis of DEGs based on the Gene Ontology database and the R package topGO.

The GO hierarchical graphs display enriched GO terms of upregulated genes in the cellular component domain (A), molecular function domain (B), and biological process domain (C) for *C. nivalis* and *C. reinhardtii*. The figure legend is in the lower-left corner of section (A).


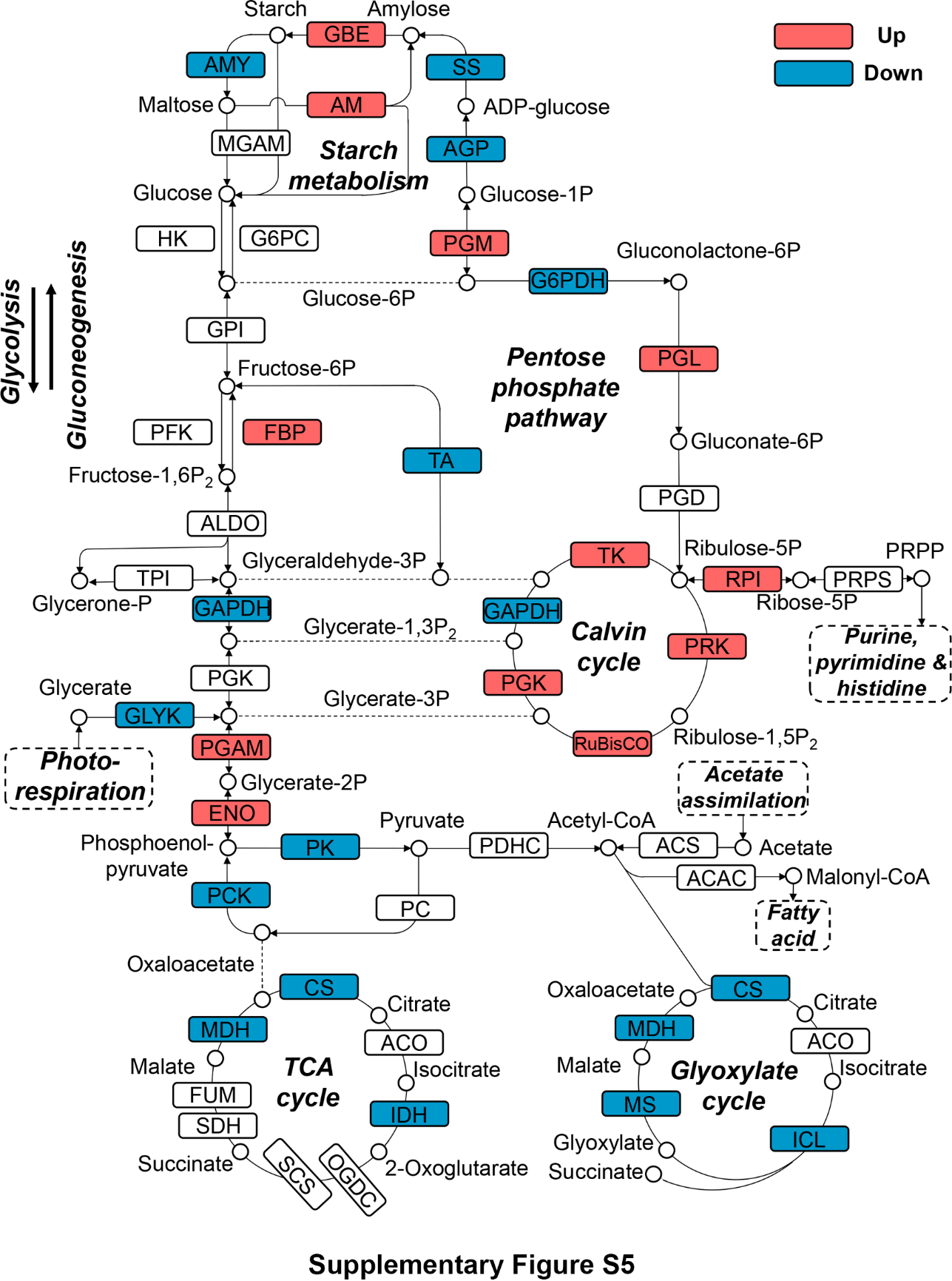


# Supplementary Figure S5. Differentially expressed genes in central carbon metabolism in *C. reinhardtii*.

Arrows represent the direction of reactions. Enzymes are marked by solid rectangles, and substrates and products are marked by circles. Dashed rectangles indicate pathways connected to carbon metabolism. Upregulated and downregulated genes are indicated by red and blue, respectively. ACAC: acetyl-CoA carboxylase / biotin carboxylase. ACO: aconitate hydratase. AGP: glucose-1-phosphate adenylyltransferase. ALDO: fructose-bisphosphate aldolase. AM: amylomaltase (4-alpha-glucanotransferase). AMY: beta-amylase. CS: citrate synthase. ENO: enolase. FBP: fructose-1,6-bisphosphatase. FUM: fumarate hydratase. G6PC: glucose-6-phosphatase. G6PDH: glucose-6-phosphate dehydrogenase. GLYK: glycerate 3-kinase. GPI: glucose-6-phosphate isomerase. HK: hexokinase. ICL: isocitrate lyase. IDH: isocitrate dehydrogenase. MDH: malate dehydrogenase. MGAM: maltase-glucoamylase. MS: malate synthase. OGDC: 2-oxoglutarate dehydrogenase complex. PC: pyruvate carboxylase. PCK: phosphoenolpyruvate carboxykinase. PDHC: pyruvate dehydrogenase complex. PFK: phosphofructokinase. PGAM: phosphoglycerate mutase. PGD: 6-phosphogluconate dehydrogenase. PGK: phosphoglycerate kinase. PGL: 6-phosphogluconolactonase. PGM: phosphoglucomutase. PK: pyruvate kinase. PRK: phosphoribulokinase. PRPP: phosphoribosylpyrophosphate. PRPS: ribose-phosphate pyrophosphokinase (PRPP synthase). RPI: ribose 5-phosphate isomerase. RuBisCO: ribulose-bisphosphate carboxylase. SCS: succinyl-CoA synthetase. SDH: succinate dehydrogenase. SS: starch synthase. TA: transaldolase. TK: transketolase. TPI: triosephosphate isomerase.


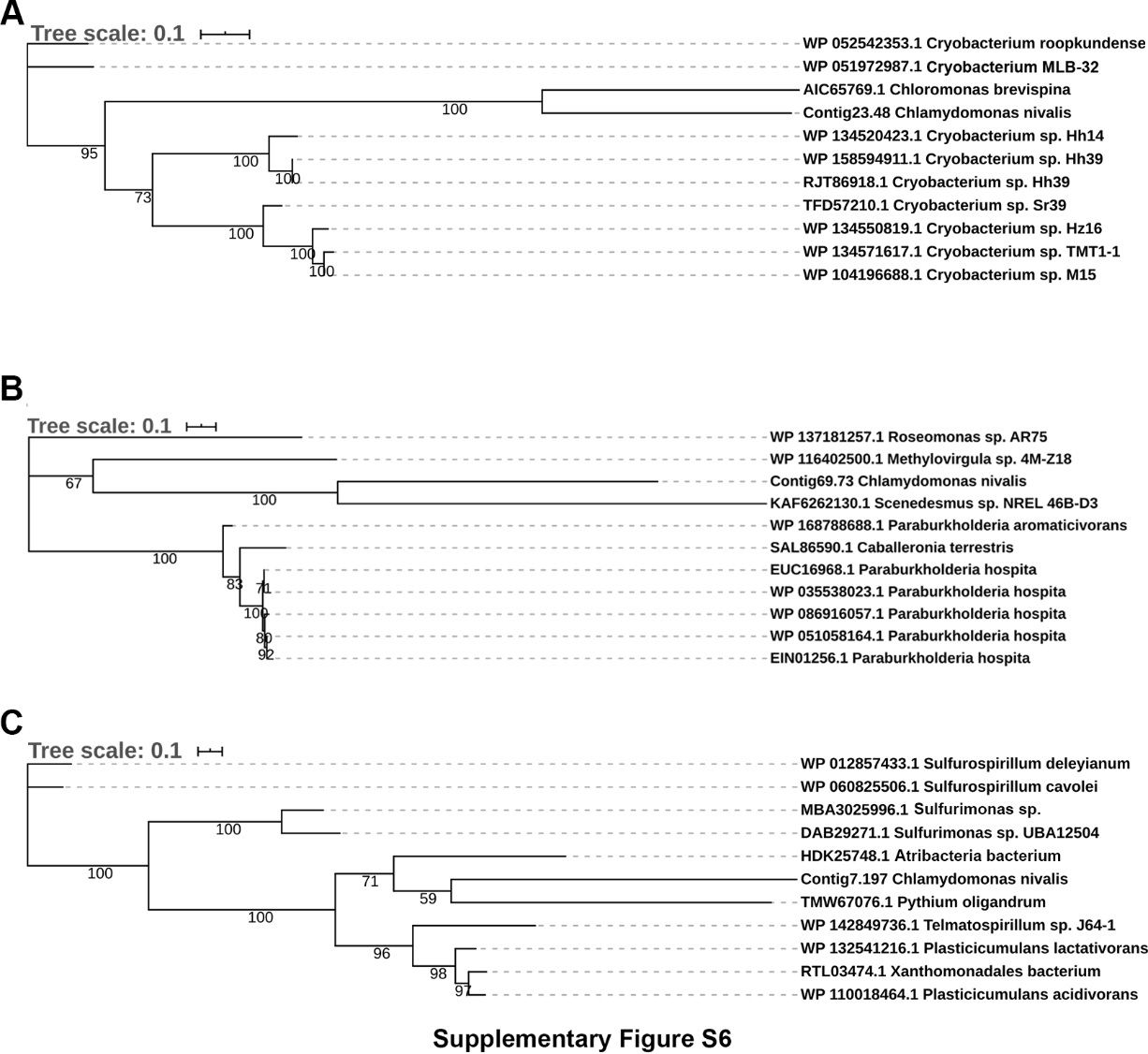


# Supplementary Figure S6. Phylogenetic trees of three certified horizontal transferred genes in *C. nivalis*.

Phylogenetic relationship of the candidate gene and 10 top hits based on Blastp is shown: (A) Contig23.48, (B) Contig 69.73, and (C) Contig7.197. The branch lengths are drawn in proportion, and the support values are shown below each branch.

# Supplementary Tables

# Supplementary Table S1. Primers used in RT-PCR.

# Supplementary Table S2A & S2B. DEGs in *C. nivalis* and *C. reinhardtii*.

# Supplementary Table S2C & S2D. Gene count matrix in *C. nivalis* and *C. reinhardtii*.

# Supplementary Table S3. DEGs inversely regulated in *C. nivalis* and *C. reinhardtii*.

# Supplementary Table S4A. DEGs uniquely existing in *C. nivalis* genome.

# Supplementary Table S4B. Candidate horizontally transferred DEGs in *C. nivalis*.

# Supplementary Table S5. Differentially expressed transcription factors in *C. nivalis* and *C. reinhardtii*.

# Supplementary Table S6. Transcription factors and interacted genes in *C. nivalis* DEGs.

# Supplementary Table S7. DEGs in *C. nivalis* homologous to *C.* ICE-L.
